# “Old Wine in a New Bottle”: Are Different Terminologies on Dual Processing Conceptually the Same?

**DOI:** 10.5964/ejop.18353

**Published:** 2026-05-29

**Authors:** C. Dominik Güss, Jaden Powell

**Affiliations:** 1 Department of Psychological and Brain Sciences, University of North Florida, Jacksonville, FL, USA; Stanford University, Stanford, USA

**Keywords:** dual-process theory, confusion with terminology, jangle fallacy, neology effect

## Abstract

**Objectives:**

Dual processing theory is among the most influential theories in cognitive psychology. It distinguishes two kinds of human thinking: one that is fast, intuitive, and effortless; and the other that is slow, deliberate, and effortful. Interestingly, these two types of human thinking have been described using many different terms by various researchers. How similar or different are these terms and related constructs?

**Methods:**

We analyzed the dual-processing literature, conducted a thought experiment, and analyses using the machine learning model BERT (SCEPTER) to compare the similarity and difference between the terms and constructs.

**Results:**

Findings show that, though these terms are lexically different, what researchers describe is, at the core, very similar. The various terms are ‘old wine in a new bottle.’

**Conclusions:**

We provide possible reasons for this coinage of terminology and present a rationale for careful deliberation before introducing new terms.

Expressing ideas is a fundamental aspect of human language. In scientific literature, researchers investigate new areas and coin new terms to describe concepts. These new terms, however, sometimes rather obfuscate ideas than clarify them, because they often overlap greatly with existing terms. This phenomenon has been called the “neology effect” (from neologisms, Pecman, 2012) or the “jangle fallacy” (Kelley, 1927). The jangle fallacy stands for different terms referring to the same underlying phenomenon. Yet, we often treat two different terms as if they were different at their core.

Scientific readability has steadily decreased, as measured by syllables per word, words per sentence, and the prevalence of ‘difficult’ words, partly due to the growing use of scientific jargon. This creates a vocabulary that is almost exclusively used and understood by scientists but is less readable by non-specialists or the public. It is especially problematic when this vocabulary does not promote the science it is attached to. The introduction of new complex expressions that are analogous to conventional constructs often masquerades as scientific progress without contributing to the conceptual growth of a field. In the literature, this phenomenon is often called the jangle fallacy, i.e., researchers mistakenly assume two essentially identical constructs are different simply because they have different labels (e.g., Friedrich et al., 2025; Kelley, 1927). Research methods, however, would allow us to assess the construct overlap — such as examining shared variance between the constructs assessed, correlational analyses, Factor analyses, and extrinsic convergent validity analyses (e.g., Gonzalez et al., 2021).

We will discuss the jangle fallacy, referring to dual process theory as an example. The theory refers to human cognition as the combined effort of two, typically opposing, processes, such as intuitive vs. deliberate or unconscious vs conscious. In Table 1, we present terms used by researchers, distinguishing the two parts of various renditions of dual process theory, the original source, and a definition clarifying the meaning of the two terms.

**Table 1:** Dual-Process Theory of Cognition Describing Human Cognitive Functions as Two Independent Parts or Processes (in chronological order)

Type 1 /System 1	Type 2/ System 2	Authors	Verbatim definition (Translated in some)	Paraphrased definition used in SCEPTER model
Erkenntnisse a posteriori. Reasoning derived from experience. Empirical Knowledge.	Erkenntnisse a priori. Reasoning prior to any experience. Pure knowledge.	Kant (1781)	“By the term “knowledge à priori,” therefore, we shall in the sequel understand, not such as is independent of this or that kind of experience, but such as is absolutely so of all experience. Opposed to this is empirical knowledge, or that which is possible only à posteriori, that is, through experience. Knowledge à priori is either pure or impure. Pure knowledge à priori is that with which no empirical element is mixed up. For example, the proposition, “Every change has a cause,” is a proposition à priori, but impure, because change is a conception which can only be derived from experience.” (Kant, 1965, p. 1).	A **posteriori knowledge** is knowledge that is derived from empirical observation and experience. It is knowledge that is gained through the senses and the accumulation of evidence. A **priori knowledge** is independent of experience; it is derived through reasoning or logic alone.
Unconscious (Ucs)	Conscious (Cs)	Freud (1915a, 1915b)	“We may say that in general a psychical act goes through two phases as regards its state, between which is interposed a kind of testing (censorship). In the first phase the psychical act is unconscious and belongs to the system Ucs.; if, on testing, it is rejected by the censorship, it is not allowed to pass into the second phase; it is then said to be ‘repressed’ and must remain unconscious. If, however, it passes this testing, it enters the second phase and thenceforth belongs to the second system, which we will call the system Cs. But the fact that it belongs to that system does not yet unequivocally determine its relation to consciousness. It is not yet conscious, but it is certainly capable of becoming conscious (to use Breuer's expression)^1^ -that is, it can now, given certain conditions, become an object of consciousness without any special resistance. In consideration of this capacity for becoming conscious we also call the system Cs. the ‘preconscious’. If it should turn out that a certain censorship also plays a part in determining whether the preconscious becomes conscious, we shall discriminate more sharply between the systems Pcs. and Cs. [Cf. p. 191 f.]. For the present let it suffice us to bear in mind that the system Pcs. shares the characteristics of the system Cs. and that the rigorous censorship exercises its office at the point of transition from the Ucs. to the Pcs. (or Cs.).” (Freud, 1915b, p. 172), (Freud, 2016, p. 15).	First, ideas and impulses begin in the unconscious, where they exist outside of our awareness. The **conscious** system is where thoughts are fully accessible to awareness.
Lower-order reasoning	Higher-order reasoning	Bloom (1956) and others	“The outcomes emphasized in those assignments may continue to be primarily knowledge and memory processes — the lowest level of the cognitive taxonomy (Bloom, 1956)”, (Treffinger 1975, p. 49). “Higher order thinking is nonalgorithmic. That is, the path of action is not fully specified in advance. Higher order thinking tends to be complex. The total path is not “visible” (mentally speaking) from any single vantage point. Higher order thinking often yields multiple solutions, each with costs and benefits, rather than unique solutions. Higher order thinking involves nuanced judgment and interpretation. Higher order thinking involves the application of multiple criteria, which sometimes conflict with one another. Higher order thinking often involves uncertainty. Not everything that bears on the task at hand is known. Higher order thinking involves self-regulation of the thinking process. We do not recognize higher order thinking in an individual when someone else “calls the plays” at every step. Higher order thinking involves imposing meaning, finding structure in apparent disorder. Higher order thinking is effortful. There is considerable mental work involved in the kinds of elaborations and judgments required.” (Resnick, 1987, p. 3). “Higher order thinking skills are grounded in lower order skills such as discriminations, simple application and analysis, and cognitive strategies and are linked to prior knowledge of subject matter content. Appropriate teaching strategies and learning environments facilitate their growth as do student persistence, self-monitoring, and open-minded, flexible attitudes.” (King et al., 2018, p. 1).	**Lower-order thinking** involves basic cognitive processes such as remembering, understanding, and applying information. **Higher-order thinking** refers to complex reasoning skills such as analyzing, evaluating, and creating. It requires critical thinking, problem-solving, and effortful judgment.
Automatic processing	Controlled processing	Schneider and Shiffrin (1977)	“Automatic processing is learned in long-term store, is triggered by appropriate inputs, and then operates independently of the subject’s control.” (Schneider & Shiffrin, 1977, p. 51). “Controlled processing is a temporary activation of nodes in a sequence that is not yet learned. It is relatively easy to set up, modify, and utilize in new situations. It requires attention, uses up short-term capacity, and is often serial in nature.” (Schneider & Shiffrin, 1977, p. 51). “Controlled processing is used to facilitate long-term learning of all kinds, including automatic processing.” (Schneider & Shiffrin, 1977, p. 51).	**Automatic processing** refers to mental operations that have been learned and stored in long-term memory. Once triggered by the right inputs, these processes run unconsciously, requiring little to no attention. **Controlled processing** involves conscious, effortful mental activity. It uses short-term memory, happens in a serial fashion, and is flexible—easily adapted to new tasks or goals.
System 1	System 2	Stanovich (1999), Kahneman (2011)	“System 1 is viewed as encompassing primarily the processes of interactional intelligence. It is automatic, largely unconscious, and relatively undemanding of computational capacity. Thus, it conjoins properties of automaticity and heuristic processing as these con-structs have been variously discussed in the literature. System 2 conjoins the various characteristics that have been viewed as typifying controlled processing. System 2 encompasses the processes of analytic intelligence that have traditionally been studied in psychometric work and that have been examined by information-processing theorists trying to uncover the computational components underlying psychometric intelligence.” (Stanovich, 1999, p. 144). “System 1: operates automatically and quickly with little effort and no sense of voluntary control” (Kahneman, 2011, p. 20). System 2: “Allocates attention to the effortful mental activities that demand it, including complex computations. The operations of system 2 are often associated with the subjective experience of agency, choice, and concentration” (Kahneman, 2011, p. 20).	**System 1** is fast, automatic, and intuitive, operating with little to no effort. **System 2** is slow, deliberate, and conscious, requiring intentional effort.
Intuitive	Deliberate	Kahneman (2003)	“The first section, Intuition and Accessibility, distinguishes two generic modes of cognitive function: an intuitive mode in which judgments and decisions are made automatically and rapidly and a controlled mode, which is deliberate and slower.” (Kahneman, 2003, p. 1)	**Intuitive** decisions are fast, automatic, and are largely unconscious. **Deliberate** decisions are slow, controlled, rational, and conscious.
Type 1 processing	Type 2 processing	Evans and Wason (1976); Evans (2009)	“We maintain that the processes which underlie such behavior (Type 1 processes) are not generally available to introspection. The other kind of process (Type 2 process) operates at a conscious level, and hence may be reported by the subject as the reason for his performance” (Evans & Wason, 1976, p. 479). “A minimal definition of the difference is that Type 1 processes are fast, automatic, low effort, and have a high processing capacity; and that Type 2 processes are slow, controlled, high effort, and have a high processing capacity.” (Evans 2009, p. 33).	**Type 1** processes are holistic, automatic, relatively undemanding of cognitive capacity, relatively fast, acquisition by personal experience, parallel, implicit, often largely unconscious or preconscious. **Type 2** processes are analytic, controlled, capacity demanding, relatively slow, acquisition by culture and formal tuition, sequential, explicit, and often conscious. (Stanovich et al., 2014).
Heuristic Processing	Analytic processing	Evans (1984)	“The term ‘heuristic’ in this paper refers to pre-attentive processes whose functions to select ‘relevant’ information for analytic processing. It determines what judgements are made about, rather than the way in which they are made” (Evans, 1984, p. 452). “It appears that any analytic judgmental or reasoning process is based only on features of the task content which have been encoded as ‘relevant’ to the task.” (Evans, 1984, p. 460).	The **heuristic system** responds automatically and rapidly to the holistic properties of stimuli. It is biased toward judgments based on overall similarity to stored prototypes. The **analytic system** processes information in terms of the internal structure of stimuli and uses systematic rules that operate on the components of stimuli, rather than processing in terms of holistic representations. (Kokis et al., 2002).
Recognition-primed decisions	Rational choice strategy	Klein (1999)	“The basic aspect of recognitional decision making is that people with experience can size up the situation and judge it as familiar or typical.” (Klein, 1999, p. 93). “Soelberg’s course on decision making at the MIT Sloan School of Management taught students how to perform the classical decision analysis method we can call the rational choice strategy. The decision maker: 1. Identifies the set of options. 2. Identifies the ways of evaluating these options.3. Weights each evaluation dimension. 4. Does the rating. 5. Picks the option with the highest score.” (Klein, 1999, p. 16).	**Recognitional decision making** is a fast, intuitive process in which individuals with experience rapidly assess a situation by recognizing familiar patterns and making judgments based on this familiarity. The **rational choice strategy** is a deliberate and systematic approach where decision makers identify available options, establish evaluation criteria, assign weights to these criteria, rate each option, and then select the option with the highest overall score. This analytical process involves careful attention and effort to ensure a well-reasoned decision.

Dual process theory dates back to 1781, as described in Kant’s “Critique of Pure Reason” (Kritik der reinen Vernunft). One could argue that dual process theory even dates back to the compartmentalization of the human soul, which was hypothesized by Socrates and Plato (Tripartite Soul, three parts). The first part is the emotional, hot-blooded part of the psyche (Thumos, spirited part), the second is the rational mind (Logistikon), and the third is the appetites, also called ‘the drives’ (Epithumia) (Plato, 2007). In this case, only the first two parts represent parts of dual process theory well.

Although dual process theory is among the most influential in cognitive psychology, it has also been criticized. Even though the distinction between two different kinds of intuitive and deliberate cognitive processes seems plausible, researchers argue that these different processes can be integrated into one unified theory (e.g., Kruglanski & Gigerenzer, 2011). Both intuitive and deliberate processes would follow the same rules, such as optimizing and satisficing. Which rules apply is dependent on the specific task and an individual’s memory and experience. Evidence for a unified theory comes from neuroscience (Mega et al., 2015). Participants were instructed to either intuitively or deliberately judge emotional facial expressions. Analyzing participants’ brain activations (conjunction, covariate, and direct contrast) showed common brain network activations in both kinds of processing and not distinct activations. These findings speak against a dichotomy of the processing systems and rather show support for a unified model. Additionally, other researchers criticize the lack of empirical support for dual process theories and the internal incoherence (Melnikoff & Bargh, 2018). The authors go so far as to call the dual-process theory a “convenient and seductive myth” (Melnikoff & Bargh, 2018, p. 280).

## Method and Results

### A Thought Experiment

In this section, we will conduct a brief thought experiment to illustrate the interchangeability of the terms described in Table 1. We hypothesized that, in each sentence, it is possible to substitute each of the terms used in dual process theory while the resulting sentence conveys the same meaning. The words we opposed in each sentence come from Table 1.

- “We humans have the ability to use our existing knowledge stored in long-term memory for UNCONSCIOUS PROCESSING, yet we also have the ability, in new and challenging situations, to attentively focus and with CONSCIOUS PROCESSING, create new solutions.” (ref. Table 1).
- “We humans have the ability to use our existing knowledge stored in long-term memory for AUTOMATIC PROCESSING, yet we also have the ability, in new and challenging situations, to attentively focus and with CONTROLLED PROCESSING, create new solutions.” (ref. Table 1).
- “We humans have the ability to use our existing knowledge stored in long-term memory for SYSTEM 1 THINKING, yet we also have the ability, in new and challenging situations, to attentively focus and with SYSTEM 2 THINKING, create new solutions.” (ref. Table 1).

These three examples illustrated that it is possible to substitute each term with another term from Table 1 in the same sentence without changing the meaning. The terms Type 1/Type 2 and the terms System 1/System 2 are the least self-explanatory among all the terms. In sum, as Table 1 and our thought experiment have shown, there are a multitude of terms used for the two distinct cognitive processes described in dual process theory. Yet, they describe the same processes.

### A Machine Learning Analysis for Natural Language Processing

In a mathematical step we investigated how similar or different the definitions of the dual process terms shown in Table 1 are. The Bidirectional Encoder Representations from Transformers (BERT) model is an open-source machine learning framework designed for natural language processing (GeeksforGeeks, 2024). The BERT model, introduced in 2018 by researchers at Google, employs an encoder-only architecture (Devlin et al., 2019). This suggests a primary emphasis on reading and understanding contextual inputs, like how humans would read and comprehend a sentence, rather than generating coherent outputs, unlike GPT models. The specific model we used to embed definitions is the sci-BERT-trained model known as “SCEPTER”, which has been pretrained on over 1 million scientific texts, primarily from the biomedical domain, which is no longer possible due to recent anti-scraping measures that most online journals have implemented (Beltagy et al., 2019). SCEPTER is ideal for our purposes because it does not require fine tuning and shows greater accuracy and distinction than sci-BERT (Cohan et al., 2020). It is specifically designed to understand human language with context (Beltagy et al., 2019). The definitions we used are shown in the last column in Table 1 (see details at Guess, 2025). Using the SCEPTER model, we attempted to show a degree of similarity between the definitions of the dual process terms we included, presenting some empirical evidence for our claim. The results are not infallible measures but would rather provide evidence to the claim that the dual-processing terminology is more similar than different.

**Figure 1:**
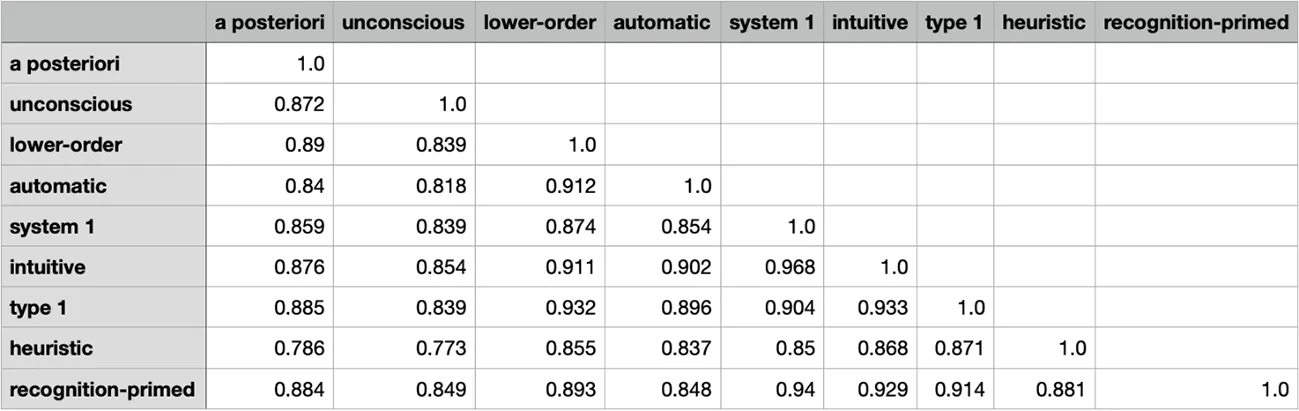
Cosine Similarity Scores for the Different Terms Used for Dual Process Theory (System 1 Part) Based on SCEPTER Embedding

**Figure 2:**
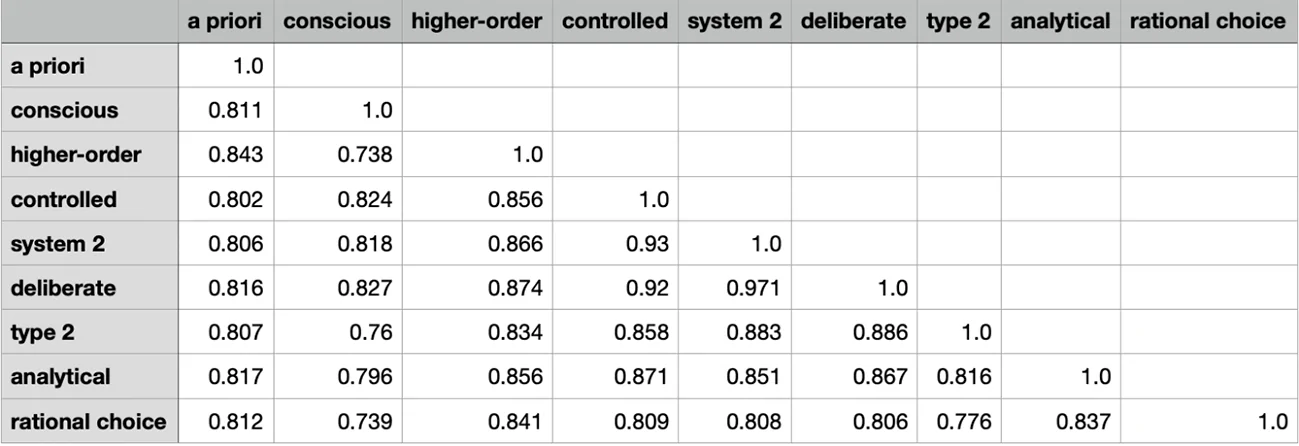
Cosine Similarity Scores for the Different Terms Used for Dual Process Theory (System 2 Part) Based on SCEPTER Embedding

Upon embedding the paraphrased definitions (Table 1) with the SCEPTER model, the terms showed a high degree of cosine similarity, further indicating their semantic similarity regardless of human comprehension. Cosine similarity scores range from -1 to 1, with, in general, any value close to 1 being considered “very similar” and any value closer to 0 being considered “not similar”. Any value below 0 is very dissimilar. However, it is important to note that bidirectional cosine similarities are far from isotropic, which means that cosine similarities are very unlikely to be negative or close to zero (Liang et al., 2021). Even though cosine similarity scores are task specific, those falling within the range of 0.4 - 0.7 can be — as a rule of thumb — considered low (Thottingal, 2025). The cosine similarity values for the definitions in our analyses ranged from 0.73 to 0.97 with 90% of our values being above .80 (Mean System 1 Internal Similarity: 0.87, Mean System 2 Internal Similarity: 0.83), indicating a strong degree of similarity between nearly all terms we consider related to System 1 or System 2, respectively (see Figure 1 and Figure 2). It is noteworthy that some terms are concrete, and others (System 1 and Type 1) are abstract summary labels. Definitions excluded direct references to terms to reduce false positive errors (e.g., ‘conscious’ was removed from its own definition “The *conscious* system is where thoughts are fully accessible to awareness”). We only compared fast/ unconscious together and then slow/ conscious together, but not fast with slow or conscious with unconscious because of linguistic and contextual overlap. In research papers describing dual process terms, definitions and usage of opposing words related to them are used in proximity to terms with opposing definitions, which unfairly weights cross cosine similarity.

### Three Possible Explanations

To summarize, using the SCEPTER model, our findings using construct definitions resulted in very high semantic similarity. For System 1, the 10 terms with the highest degrees of cosine similarity (all above 0.90) were automatic — lower-order, intuitive — lower-order, intuitive — automatic, intuitive — System 1, Type 1 — lower-order, Type 1 – System 1, Type 1 — intuitive, recognition-primed — system 1, recognition-primed — intuitive, and recognition-primed — Type 1; For System 2, the three terms with the highest degree of cosine similarity (all above 0.90) were System 2 — controlled, deliberate — controlled, and deliberate — System 2.

To explain the phenomenon of ideologically similar terminology, we asked ourselves why scientists create new terms conceptually analogous to conventional terms. One possible explanation is the evolution of scientific language. Kuhn (1962) argues that as science evolves, changes in fundamental theories drive shifts in meaning, which, in turn, drive a shift in terminology. “What were ducks in the scientist’s world before the revolution are rabbits afterwards” (Kuhn 1962, p. 111). Similarly, Chomsky (2000) and Wittgenstein (1953) argue that common words and the use of existing words change over time as culture changes their meanings. “For a large class of cases — though not for all — in which we employ the word ‘meaning’, it can be defined thus: the meaning of a word is its use in the language” (Wittgenstein 1953, p. 20). Terms provided in Table 1 span a period of over 200 years (1781 to 2011). Given this time frame, it is plausible that the fundamental theories that make up dual-process theory and, therefore, the language describing the theory have developed and adjusted according to this view. These language shifts could reflect changes in technology, psychology, and society.

Another possible reason for creating new terms is that researchers change terms and theories when there are conflicts between existing theories and predictions and acquired knowledge (Kuhn, 1962). This occurs when a subtlety related to an existing theory is not expressed adequately and is worth highlighting in a newly developed term (different meanings associated with different terms, e.g., Güss, 2011). For example, researchers might have been dissatisfied with a focus on the conscious and unconscious aspects of cognition and changed focus from ‘conscious processing’ and ‘unconscious processing’ to ‘heuristic processing’ and ‘analytical processing’, which highlight the speed and type of cognitive processing (quick and based on experience vs. slow and thoughtful). Leahey (2018) expands on the dissatisfaction of academic psychologists with psychoanalysis and the related terms conscious/unconscious. To be fair, research related to every term might address a nuance or specific aspect of the dual process theory in more depth than other dual process theories and studies.

A third possible reason is behavioral but related to the pressures and competition in the university context. Researchers at most research universities are under enormous pressures, for example, to “publish or perish”, get cited, bring in grant monies, get excellent student evaluations for their teaching (Martinson, 2011; Martinson et al., 2009), and get tenure. Researchers are incentivized to coin new terms to gain recognition, as they will be associated with their names (see, e.g., Pecman, 2012; neologisms in the field of climate change, Zella et al., 2025). The researchers can argue that their term describes an idea that contributes to science when, under scrutiny, existing terms and their related concepts adequately describe the theory. Fame or recognition as a reward for coining a “revolutionary” term, irrespective of its conceptual novelty, outweighs the drawback of contributing to terminological inflation. This problem in the field of psychology has been described as the toothbrush problem (Mischel, 2008, who forgot the original source): “Psychologists treat other peoples’ theories like toothbrushes — no self-respecting person wants to use anyone else’s”.

## Discussion

We have listed several different terms used for dual process theory in Table 1 and provided possible explanations as to why there are multiple terms used to express the same phenomenon.

We urge researchers to be cautious when coining new terms and not fall for the jangle fallacy. Just as Chomsky argues for abandoning vague commonsense terms for concise conceptualization in science (Chomsky, 2000), we should also abandon redundant or semi-distinct terms in academic discourse that do not offer useful conceptual gains (see also Pekrun, 2023). If even scientists in the same field find certain terminology difficult to understand, one can only imagine how difficult these terms may be for scientists in other fields and for lay people.

Psychologists are well equipped with statistical methodologies, and these can be used to test the overlap, distinctiveness, and validity of constructs (Hodson, 2021; Strand et al., 2020). Procedures such as applying multiple measures in research to calculate convergent *and* discriminant construct validity, analyzing correlations, testing for measurement issues, assessing the fit of the data with the theory, constraining generality statements to a specific population and context, make this a transparent approach.

Going back to the terminology, both Stanovich (1999) and Evans (2009), for example, argue for the use of general terms that encompass and distinctly separate all components of dual process theory. However, the terms they created are not as effective as they could be. Stanovich’s “System 1” and “System 2” and Evan’s “Type 1” and “Type 2” are terms that lack intrinsic meaning without an accompanying conceptual framework. As a result, non-specialists cannot intuitively understand the ideas these terms are intended to convey. Since a word is only as meaningful as its usage, scientific constructs that are easier to understand are more likely to be adopted and carry greater influence (Wittgenstein, 1953). A goal for term development in the field of cognitive psychology — and in other disciplines that struggle with analogous terminology — should be to create general expressions that are intuitively meaningful to a non-specialist while precise enough to avoid ambiguity within and beyond the field.

If the trend in scientific fields is to refine language by stripping terms of ambiguity and tying them to specific theories, then the creation of new terms should be justified by conceptual necessity, not because of trend or novelty for novelty’s sake. Once an unambiguous scientific term has been established and its related conceptual necessity fulfilled, further excess terminology risks inflating the field’s lexicon without deepening understanding. This not only reduces conceptual clarity but also makes some scientific findings inaccessible to out-of-field researchers or readers, increasing domain exclusivity. In a similar way, researchers have criticized the many “disjointed efforts that go into the numerous constructs and measures proliferating in the field” of psychology (e.g., Anvari et al., 2025, p. 839).

Potentially, this lack of common terminology also leads to exponential research output making it even more difficult for any one person, expert or not, to fully grasp what is going on. The search term “dual process theory” showed 4,500,000 results in Google Scholar — at the time of writing this article. This number does not even include other potential publications with similar search terms. We are increasingly relying on meta-analyses and reviews to keep current, but potentially forgetting the 6^th^ or 7^th^ search term could result in big gaps in our knowledge.

Wittgenstein (1953) argues that language is like a game where the socio-cultural context creates the rules. In this game, new words only gain traction if they are widely used within this context. In a way, all the researchers mentioned in Table 1 “won” the game because their terms have been widely used and adopted. However, if these terms spawn from anything other than a conceptual necessity, they risk cluttering the lexicon and consequently hiding their findings from other “players”. Scientists have the means to shape the vocabulary of scientific and public discourse, and with that comes responsibility. We urge scholars to prioritize lexical accessibility and theoretical necessity in naming. A well-chosen term should not only withstand scholarly scrutiny but serve as a bridge between disciplines and between science and the public.

## Supplementary Materials

**Table:** 

Type of supplementary material	Availability/Access
Data	
No data provided.	—
Code	
Python code	Guess (2025)
Material	
No study materials provided.	—
Study/Analysis preregistration	
Study was not preregistered	—
Other	
Method and data procedure.	Guess (2025)

## Data Availability

All data is reported and shown in the article. Details to code and method are available in the OSF repository at Guess (2025).

## References

[r1] Anvari, F., Alsalti, T., Oehler, L. A., Hussey, I., Elson, M., & Arslan, R. C. (2025). Defragmenting psychology. Nature Human Behaviour, 9, 836–839. 10.1038/s41562-025-02138-0 40102675

[r2] Beltagy, I., Lo, K., & Cohan, A. (2019). SciBERT: A pretrained language model for scientific text. In S. Padó & R. Huang (Eds.), *Proceedings of the 2019 Conference on Empirical Methods in Natural Language Processing and the 9th International Joint Conference on Natural Language Processing* (EMNLP-IJCNLP) (pp. 3606–3611). Association for Computational Linguistics. https://aclanthology.org/D19-3.pdf

[r3] Bloom, B. S. (1956). *Taxonomy of educational objectives: The classification of educational goals* (1^st^ ed.). Longman Group.

[r4] Chomsky, N. (2000). *New horizons in the study of language and mind*. Cambridge University Press.

[r5] Cohan, A., Feldman, S., Beltagy, I., Downey, D., & Weld, D. S. (2020). SPECTER: Document-level representation learning using citation-informed transformers. In D. Jurafsky, J. Chai, N. Schluter & J. Tetreault (Eds.), *Proceedings of the 58^th^ Annual Meeting of the Association for Computational Linguistics* (pp. 2270–2282). Association for Computational Linguistics. https://aclanthology.org/2020.acl-main.pdf

[r6] Devlin, J., Chang, M. W., Lee, K., & Toutanova, K. (2019, June). Bert: Pre-training of deep bidirectional transformers for language understanding. In *Proceedings of the 2019 Conference of the North American Chapter of the Association for Computational Linguistics: Human Language Technologies, Vol. 1*, (pp. 4171–4186).

[r7] Evans, J. S. B. T. (1984). Heuristic and analytic processes in reasoning. British Journal of Psychology, 75(4), 451–468. 10.1111/j.2044-8295.1984.tb01915.x

[r8] Evans, J. S. B. T. (2009). *How many dual-process theories do we need? One, two, or many?* In J. Evans & K. Frankish (Eds.), *In two minds: Dual processes and beyond* (pp. 33–54). Oxford University Press. 10.1093/acprof:oso/9780199230167.003.0002

[r9] Evans, J. S. B. T., & Wason, P. C. (1976). Rationalisation in a reasoning task. British Journal of Psychology, 67(4), 479–486. 10.1111/j.2044-8295.1976.tb01536.x

[r10] Freud, S. (1915 a). I. Das Unbewußte [The Unconscious]. Internationale Zeitschrift für Psychoanalyse, 3, 189–203.

[r11] Freud, S. (1915b). The unconscious. In J. Strachey (Ed. & Trans.), *The standard edition of the complete psychological works of Sigmund Freud* (Vol. 14, pp. 159–215). Hogarth Press. (Original work published 1915).

[r12] Freud, S. (2016). *Das Unbewusste* [*The Unconscious*] Reclam.

[r13] Friedrich, J., Fischer, M. H., & Raab, M. (2025). Issues in grounded cognition and how to solve them — The minimalist account. Journal of Cognition, 8(1), 31. 10.5334/joc.444 40290452 PMC12023178

[r14] GeeksforGeeks. (2024, December 10). *BERT Model — NLP*. https://www.geeksforgeeks.org/explanation-of-bert-model-nlp/

[r15] Gonzalez, O., MacKinnon, D. P., & Muniz, F. B. (2021). Extrinsic convergent validity evidence to prevent jingle and jangle fallacies. Multivariate Behavioral Research, 56(1), 3–19. 10.1080/00273171.2019.1707061 31958017 PMC7369230

[r16] Guess, D. (2025). *Different terminologies for dual processing* [OSF project page containing method and data files]. OSF. https://osf.io/eq5ma/overview

[r17] Güss, C. D. (2011). Suicide terrorism: Exploring Western perceptions of terms, context, and causes. Behavioral Sciences of Terrorism and Political Aggression, 3(2), 97–115. 10.1080/19434472.2010.512152

[r18] Hodson, G. (2021). Construct jangle or construct mangle? Thinking straight about (nonredundant) psychological constructs. Journal of Theoretical Social Psychology, 5(4), 576–590. 10.1002/jts5.120

[r19] Kahneman, D. (2003). A perspective on judgment and choice: Mapping bounded rationality. The American Psychologist, 58(9), 697–720. 10.1037/0003-066X.58.9.697 14584987

[r20] Kahneman, D. (2011). *Thinking, fast and slow*. Farrar, Straus and Giroux.

[r21] Kant, I. (1965). *Immanuel Kant’s Critique of Pure Reason* (Norman Kemp Smith, Trans.). St. Martin’s Press.

[r22] Kant, I. (1781). *Kritik der reinen Vernunft* [Critique of Pure Reason]. Johann Friedrich Hartknoch.

[r23] Kelley, T. L. (1927*). **Interpretation of educational measurements*. World Book Co.

[r24] King, F. J., Goodson, L., & Rohani, F. (2018). *Higher order thinking skills: Definition, teaching strategies, assessment*. Center for Advancement of Learning and Assessment, Florida State University.

[r25] Klein, G. (1999). *Sources of power: How people make decisions*. MIT Press.

[r26] Kokis, J. V., Macpherson, R., Toplak, M. E., West, R. F., & Stanovich, K. E. (2002). Heuristic and analytic processing: Age trends and associations with cognitive ability and cognitive styles. Journal of Experimental Child Psychology, 83(1), 26–52. 10.1016/S0022-0965(02)00121-2 12379417

[r27] Kruglanski, A. W., & Gigerenzer, G. (2011). Intuitive and deliberate judgments are based on common principles. Psychological Review*, *118(1), 97–109. 10.1037/a0020762 21244188

[r28] Kuhn, T. (1962). *The structure of scientific revolutions*. University of Chicago Press.

[r29] Leahey, T. H. (2018). *A history of psychology: from antiquity to modernity* (8^th^ ed.). Pearson.

[r30] Liang, Y., Cao, R., Zheng, J., Ren, J., & Gao, L. (2021). Learning to remove: Towards isotropic pre-trained BERT embedding. *arXiv*. 10.1007/978-3-030-86383-8_36

[r31] Martinson, B. C., Crain, A. L., Anderson, M. S., & De Vries, R. (2009). Institutions’ expectations for researchers’ self-funding, federal grant holding, and private industry involvement: Manifold drivers of self-interest and researcher behavior. Academic Medicine : Journal of the Association of American Medical Colleges, 84(11), 1491–1499. 10.1097/ACM.0b013e3181bb2ca6 19858802 PMC3071700

[r32] Martinson, B. C. (2011). The academic birth rate. Production and reproduction of the research work force, and its effect on innovation and research misconduct. EMBO Reports, 12(8), 758–762. 10.1038/embor.2011.142 PMC3147269 21738219

[r33] Mega, L. F., Gigerenzer, G., & Volz, K. G. (2015). Do intuitive and deliberate judgments rely on two distinct neural systems? A case study in face processing. Frontiers in Human Neuroscience*, *9, 456. 10.3389/fnhum.2015.00456 PMC4548224 26379523

[r34] Melnikoff, D. E., & Bargh, J. A. (2018). The mythical number two. Trends in Cognitive Sciences*, *22(4), 280–293. 10.1016/j.tics.2018.02.001 29571664

[r35] Mischel, W. (2008). The toothbrush problem. *Observer, 12*. https://www.psychologicalscience.org/observer/the-toothbrush-problem

[r36] Pecman, M. (2012). Tentativeness in term formation: A study of neology as a rhetorical device in scientific papers. Terminology, 18(1), 27–58. 10.1075/term.18.1.03pec

[r37] Pekrun, R. (2023). Jingle-jangle fallacies in motivation science: Toward a definition of core motivation. In M. Bong, J. Reeve & S.-I. Kim (Eds.), *Motivation science: Controversies and insights* (pp. 52–58). Oxford University Press. 10.1093/oso/9780197662359.003.0009

[r38] Plato. (2007). *The Republic*. (D. Lee, Trans.; 2^nd^ ed.). Penguin.

[r39] Resnick, L. B. (1987). *Education and learning to think*. National Academy Press. 10.17226/1032

[r40] Schneider, W., & Shiffrin, R. M. (1977). Controlled and automatic human information processing: I. Detection, search, and attention. Psychological Review, 84(1), 1–66. 10.1037/0033-295X.84.1.1

[r41] Stanovich, K. E. (1999). *Who is rational? Studies of individual differences in reasoning*. Lawrence Erlbaum Associates Publishers.

[r42] Stanovich, K. E., West, R. F., & Toplak, M. E. (2014). Rationality, intelligence, and the defining features of Type 1 and Type 2 processing. In J. W. Sherman, B. Gawronski & Y. Trope (Eds.), *Dual-process theories of the social mind* (pp. 80–91). Guilford Press.

[r43] Strand, J. F., Ray, L., Dillman-Hasso, N. H., Villanueva, J., & Brown, V. A. (2020). Understanding speech amid the Jingle and Jangle: Recommendations for improving measurement practices in listening effort research. Auditory Perception & Cognition, 3(4), 169–188. 10.1080/25742442.2021.1903293 34240011 PMC8262528

[r44] Thottingal, S. (2025). Question-to-Question retrieval for hallucination-free knowledge access: An approach for Wikipedia and Wikidata question answering. *arXiv*. https://arxiv.org/html/2501.11301v3

[r45] Treffinger, D. J. (1975). Teaching for self-directed learning: A priority for the gifted and talented. Gifted Child Quarterly*, *19(1), 46–59. 10.1177/001698627501900109

[r46] Wittgenstein, L. (1953). *Philosophical investigations/Philosophische Untersuchungen.* Macmillan.

[r47] Zella, G., Bolderdijk, J. W., Caselli, T., & Peels-Matthey, S. (2025). The role of neologisms in the climate change debate: Can new words help to speed up social change? Wiley Interdisciplinary Reviews: Climate Change, 16(2), e70004. 10.1002/wcc.70004

